# Supplementary material for: Association between oral microbiome and seven types of cancers in East Asian population: a two-sample Mendelian randomization analysis
Source: Front Mol Biosci. 2023 Nov 21;10:1327893. doi: 10.3389/fmolb.2023.1327893 (PMC10702768; doi:10.3389/fmolb.2023.1327893)
Supplement: Supplementary file 1 [file DataSheet1.ZIP › Supplementary Materials/MR plots for tongue/tongue═╝/Colorectal cancer/pheno.2462_to_colorectal cancer_forest.pdf]

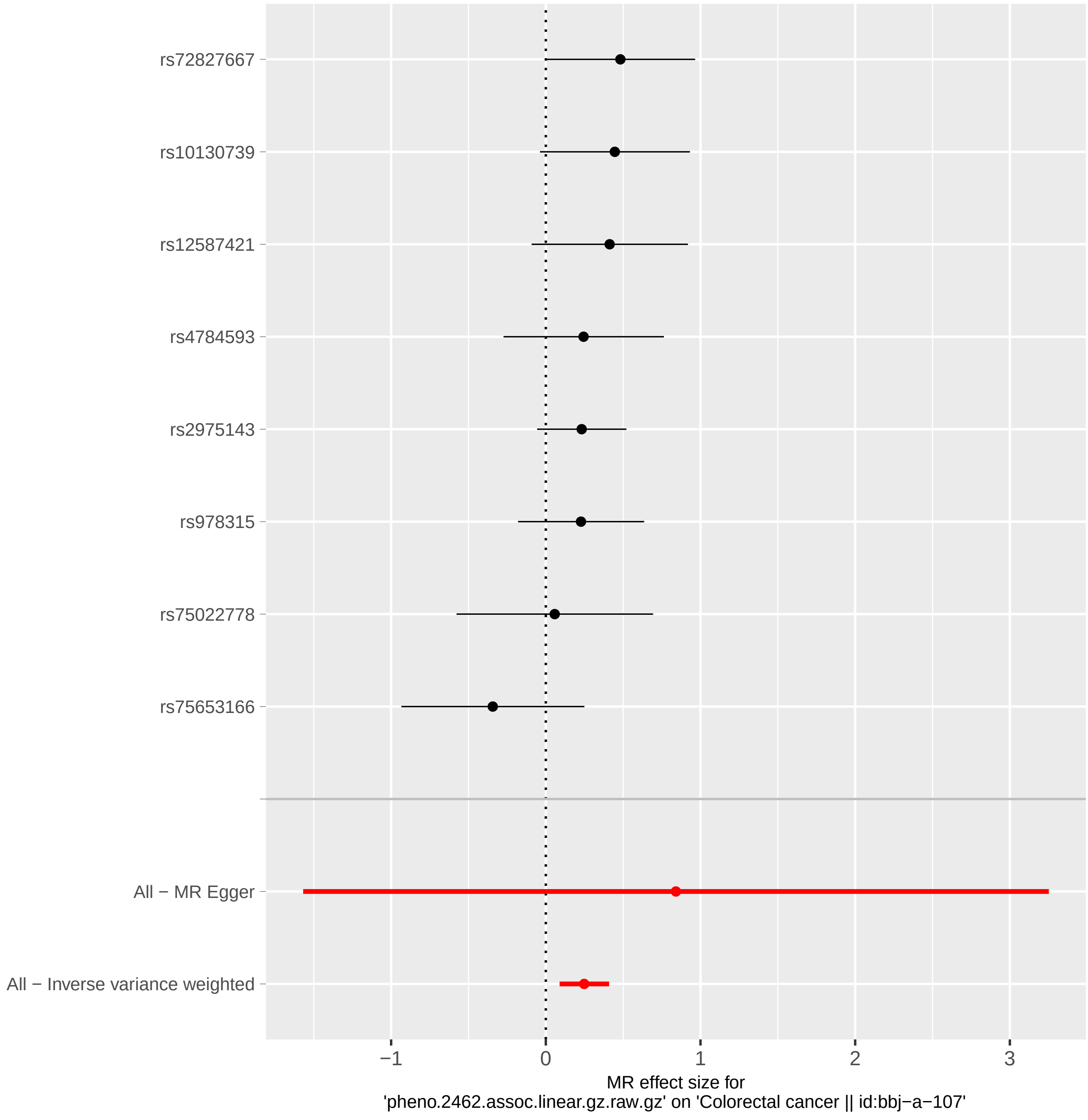
rs72827667
rs10130739
rs12587421
rs4784593
rs2975143
rs978315
rs75022778
rs75653166
All − MR Egger
All − Inverse variance weighted
−1
0
1
2
3
MR effect size for
'pheno.2462.assoc.linear.gz.raw.gz' on 'Colorectal cancer || id:bbj−a−107'
